# CD4^+^ T-Cell Activation Prompts Suppressive Function by Extracellular Vesicle-Associated MicroRNAs

**DOI:** 10.3389/fcell.2021.753884

**Published:** 2021-10-27

**Authors:** Dario Di Silvestre, Silvia Garavelli, Claudio Procaccini, Francesco Prattichizzo, Giulia Passignani, Veronica De Rosa, Pierluigi Mauri, Giuseppe Matarese, Paola de Candia

**Affiliations:** ^1^Istituto di Tecnologie Biomediche, Consiglio Nazionale delle Ricerche (ITB-CNR), Milan, Italy; ^2^Laboratorio di Immunologia, Istituto di Endocrinologia e Oncologia Sperimentale, Consiglio Nazionale delle Ricerche (IEOS-CNR), Naples, Italy; ^3^Unitá di Neuroimmunologia, IRCCS Fondazione Santa Lucia, Roma, Italy; ^4^IRCCS MultiMedica, Milan, Italy; ^5^Treg Cell Laboratory, Dipartimento di Medicina Molecolare e Biotecnologie Mediche, Universitá Degli Studi di Napoli “Federico II”, Naples, Italy

**Keywords:** exosomes, extracellular vesicles, microRNA, CD4^+^ T cells, cell activation

## Abstract

MicroRNAs (miRNAs), small non-coding molecules targeting messenger RNAs and inhibiting protein translation, modulate key biological processes, including cell growth and development, energy utilization, and homeostasis. In particular, miRNAs control the differentiation, survival, and activation of CD4**^+^** T conventional (Tconv) cells, key players of the adaptive immunity, and regulate the physiological response to infections and the pathological loss of immune homeostasis in autoimmunity. Upon T-cell receptor (TCR) stimulation, the described global miRNA quantitative decrease occurring in T cells is believed to promote the acquisition of effector functions by relaxing the post-transcriptional repression of genes associated with proliferation and cell activity. MiRNAs were initially thought to get downregulated uniquely by intracellular degradation; on the other hand, miRNA secretion *via* extracellular vesicles (EVs) represents an additional mechanism of rapid downregulation. By focusing on molecular interactions by means of graph theory, we have found that miRNAs released by TCR-stimulated Tconv cells are significantly enriched for targeting transcripts upregulated upon stimulation, including those encoding for crucial proteins associated with Tconv cell activation and function. Based on this computational approach, we present our perspective based on the following hypothesis: a stimulated Tconv cell will release miRNAs targeting genes associated with the effector function in the extracellular space in association with EVs, which will thus possess a suppressive potential toward other Tconv cells in the paracrine environment. We also propose possible future directions of investigation aimed at taking advantage of these phenomena to control Tconv cell effector function in health and autoimmunity.

## Introduction

CD4^+^ T conventional (Tconv) cells represent the master orchestrators of the adaptive immunity. Naïve Tconv cells respond to T-cell receptor (TCR) stimulation (i.e., antigen encounter), costimulatory molecules, and cytokines by undertaking clonal expansion and engaging effector and memory functions (Bluestone et al., 2009). The transcriptional circuits of CD4^+^ T cells (Tconv but also the T regulatory subset) are highly responsive to regulation by microRNAs, which cooperate with epigenetic remodeling and lineage-restricted transcription factors to sculp the transcriptome and direct the functional outputs (modulation of energy metabolism, proliferation, and cytokine production) (Garavelli et al., 2018). MicroRNAs (miRNAs) are small (∼22 nucleotides in length), non-coding RNAs, transcribed by RNA polymerase II as longer RNAs called “pri-miRNAs” (Lee et al., 2004). The first steps of maturation involve the sequential action of two endoribonucleases, Drosha and Dicer, to generate the miRNA duplex (containing the miRNA paired to its passenger strand), which is loaded into an Argonaute (Ago) protein; then, the expulsion of the miRNA passenger strand leads to the formation of the mature RNA-induced silencing complex (RISC). Once loaded into the RISC, the miRNA pairs to sites usually within the 3′ untranslated region of messenger (m)RNAs, causing mRNA decay and block of translation (Bartel, 2018).

All eukaryotes share the miRNA-dependent post-transcriptional gene expression regulation, which possibly evolved from the ancient RNA interference (RNAi) process, and the majority of human mRNAs are known to be targeted by miRNAs, potentially implicating these molecules in all cells and all physio/pathological processes (Friedman et al., 2009). The general role of miRNAs in CD4^+^ T cells is revealed by experimental conditions in which all miRNA action has been blocked in these cells: either Dicer or Drosha deficiency demonstrates a significant skew toward a pro-inflammatory phenotype leading to spontaneous inflammation/autoimmunity (Cobb et al., 2005; Muljo et al., 2005; Chong et al., 2008). Similarly, Ago-deficient T cells, which also suffer from miRNA depletion, are likewise predisposed to differentiate into effector cytokine-releasing cells (Bronevetsky et al., 2013). From these studies, it seems reasonable to hypothesize that miRNA action generally restrains the acquisition of effector functions by CD4^+^ T cells, possibly repressing transcripts that propel Tconv cell activation (Garavelli et al., 2018). Consistently with this hypothesis, TCR stimulation is known to lead to a global miRNA level decrease that accompanies a net increase of total RNA yield per cell and the induction of a plethora of transcripts (Bronevetsky et al., 2013). This miRNA decrease is believed to depend on both pri-miRNA transcription drop and a decline in RISC activity (Bronevetsky et al., 2013).

Through robust proliferation, T cells may also passively dilute abundant miRNAs simply by cell division, but this mechanism cannot occur in the first hours of activation, i.e., before mitosis actually starts. An additional mechanism for the cell to rapidly downregulate miRNAs is through their disposal into the extracellular milieu *via* extracellular vesicles (EVs), membrane surrounded bodies of nanometric size (from 50 nm to 1 micron), with well-characterized biogenesis (Raposo and Stoorvogel, 2013; Zhang et al., 2014); it is the case of miR-150, whose extracellular accumulation is concomitant with intracellular downregulation and subsequent induction of its target, the transcriptional factor c-Myb, which, together with other mRNAs, critically promotes lymphocyte survival and response (de Candia et al., 2013). If this regulatory mechanism stands true for other miRNAs as well, then EVs may be enriched for molecules targeting transcripts that need to be upregulated upon T-cell activation, and thus EVs derived from a TCR-stimulated T cell may carry a “T cell suppressive cargo” in the extracellular milieu. The association between this potential biological function and T-cell activation is strengthened by the observation that unstimulated T cells are mostly inactive in EV production (Torri et al., 2017). In the present perspective, we have directly tested this hypothesis by a computational approach focusing on molecular interactions by means of graph theory (Vella et al., 2017). In addition, we combined miRNA–target interaction and protein–protein interaction (PPI) network models with the purpose of predicting the main players involved in the suppressive function potentially exerted by EV-associated miRNAs.

### MicroRNAs Released by T-Cell Receptor-Stimulated T Conventional Cells Are Significantly Enriched for Targeting Transcripts Upregulated During T-Cell Activation

**FIGURE 1:**
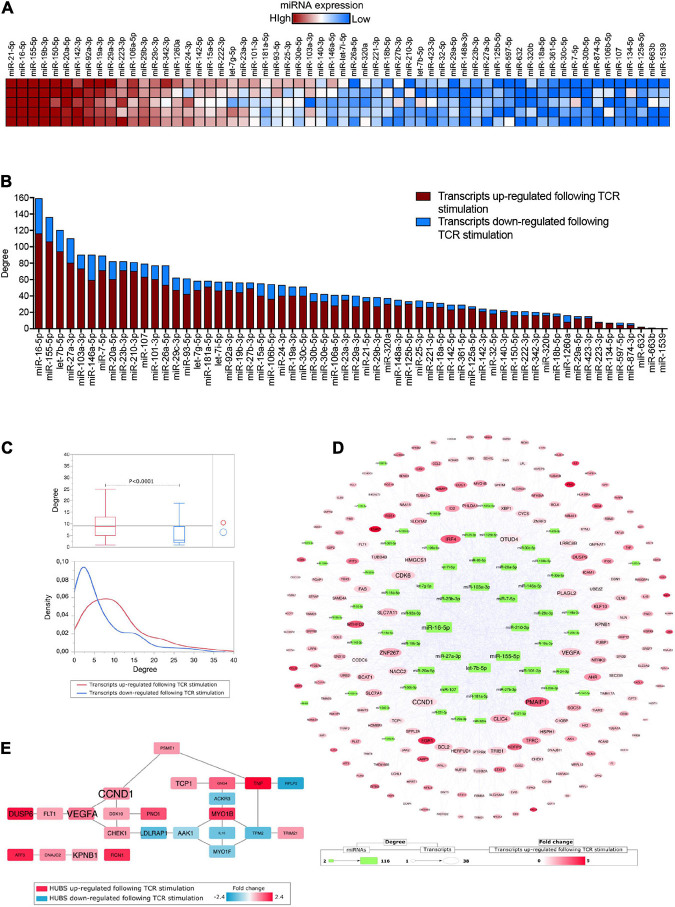
**(A)** Heatmap showing miRNA expression (*n* = 60) in extracellular vesicles released by *in vitro* TCR-stimulated Tconv cells (isolated from five healthy donors). Each molecule was normalized by miRNA global mean and ranked based on relative mean expression value from the most to the least expressed. **(B)** Bar histogram showing the number of miRNA targets (among those transcripts either upregulated, red, or downregulated, blue, following TCR stimulation) for each of the 60 detectable Tconv-derived EV-miRNAs, ranked based on target total number. **(C)** Box plots comparing the number of miRNA–target interactions (degree, upper panel) and density distribution of the interactions (lower panel) distinguishing the transcripts (targets) for being either upregulated (red) or downregulated (blue) following TCR stimulation (Student’s *t*-test, *p* < 0.0001). **(D)** MiRNAs–targets network reconstructed considering only transcripts upregulated following TCR stimulation. **(E)** Protein–protein interaction HUBS selected by considering Betweenness, Bridging, and Centroid centralities; the gene name size is proportional to the number of miRNAs targeting it.

In order to evaluate the EV-associated miRNome released by human CD4^+^ Tconv cells upon *in vitro* activation, we have isolated naïve CD4^+^CD25^–^ T cells circulating in peripheral blood of five healthy subjects and stimulated them *in vitro* with anti-CD3/anti-CD28 beads. Compared to previous analysis (Torri et al., 2017), cells were stimulated by a low concentration of beads (0.2/cell), which is able to better mimic conditions of T-cell activation *in vivo*. After 72 h, EVs were isolated from conditioned medium through size exclusion chromatography and 752 human miRNAs were profiled by quantitative RT-PCR. Sixty EV-associated miRNAs out of 752 (7.9%) were identified as detectable in Tconv cell-derived EVs, for showing a Ct <35 in at least 4/5 subjects, and were normalized by internal miRNA global mean (Figure 1A): their mean relative expression values are reported in Supplementary Table 1. Among the most expressed EV-associated miRNAs (EV-miRNAs), we spotted molecules with well-characterized immune regulatory functions, such as miR-21-5p, miR-155-5p, miR-150-5p, and miR-106a-5p (Figure 1A; Garavelli et al., 2018). MiRNA relative quantities in EVs did generally correlate with those registered at the intracellular level although the correlation index was not strong (Pearson *R* = 0.5, Supplementary Figure 1A), a result in line with previous reports (de Candia et al., 2013; Torri et al., 2017). Furthermore, miRNAs well known to be downregulated upon TCR stimulation (Rodríguez-Galán et al., 2018, 2021) demonstrated to be enriched in EVs compared with the intracellular milieu (Supplementary Figure 1B), supporting the hypothesis that miRNA disposal by EV release may represent an additional pathway of miRNA downregulation.

To evaluate the correlation between EV-miRNAs released by TCR-stimulated Tconv cells and the TCR-dependent transcriptional modulation in the same cells, we took advantage of the list of transcripts that were recently identified as differentially expressed (DE) in human peripheral blood-derived CD4^+^CD25^–^ T cells upon 12 h of TCR stimulation (anti-CD3/CD28 beads, at 0.2 beads/cell) (Procaccini et al., 2021). The list of DE transcripts is composed of 231 upregulated and 129 downregulated transcripts (total number = 360, relative mean expression values and fold changes, reported in Supplementary Table 1). By combining Tconv cell-derived EV-miRNAs and the TCR-dependent transcriptional modulation, we found that 304 out of 360 (84.4%) DE transcripts were experimental validated targets of at least one of the considered miRNAs (Supplementary Table 1). The two miRNAs targeting the highest number of DE transcripts considered (degree >120) were miR-16-5p and miR-155-5p, which were also the second and third most represented miRNA in EVs (with mean relative quantities of +3.90 for both miRNAs); on the other hand, miR-1539 and miR-663b were those with the lowest number of targets (<2) and also the least represented in EVs (with mean relative quantities of −2.85 and −2.77, respectively) (Figure 1B and Supplementary Table 1). Beside the absolute number of targeted mRNAs, the great majority of these mRNAs are actually upregulated upon activation (red in Figure 1B, mean% equal to 78.33 for miRNAs with >2 targets), suggesting that the resulting overall function of EV-associated miRNome is to actually suppress activation-dependent transcriptional circuits. In further support of this hypothesis, we found that, on average, DE transcripts upregulated following TCR stimulation are targeted by a number of EV-miRNAs, which is double that comprising of miRNAs targeting downregulated transcripts (Student’s *t*-test, *p* < 0.0001, Figure 1C).

### Crucial Genes Associated to T Conventional Cell Activation and Function Are Targeted by Multiple Extracellular Vesicle-MicroRNAs Released by T Conventional Cells

By reconstructing a miRNAs–targets network based on upregulated DE transcripts following TCR stimulation in Tconv cells, we were able to reveal both EV-miRNAs and intracellular targets with the highest grade of connections (Figure 1D). In particular, among the transcripts targeted by the highest numbers of considered EV-miRNAs (degree >30), we spotted CCND1 (G1/S-specific cyclin-D1), PMAIP1 (Phorbol-12-myristate-13-acetate-induced protein 1), CDK6 (Cyclin-dependent kinase 6), and VEGFA (Vascular endothelial growth factor A), all critically involved in cytokine-mediated signaling pathway, cell cycle/proliferation, and apoptotic processes (Figure 1D and Supplementary Table 1). Another highly targeted mRNA encodes for ZNF267 (Zinc finger protein 267), which belongs to the family of Kruppel-like transcription factors and regulates fundamental biological processes such as development, proliferation, and differentiation; intriguingly, among the 31 Tconv cell-derived EV-miRNAs targeting this transcript, miR-23a-3p and miR-23b-3p were already reported to regulate it with an EV-mediated mechanism (Lu et al., 2015). Interferon regulatory factor 4 (IRF4), a transcription factor critically involved in maturation and differentiation of naïve CD4^+^ T cells into effector cells (Th1, Th2, Th9, Th17, and T reg subsets) (Huber and Lohoff, 2014), TFRC (Transferrin Receptor, necessary for cellular iron uptake) whose upregulation on the surface of T cells is among the earliest and provides necessary cues for T cell activation and proliferation (Batista et al., 2004), Cytosolic Branched Chain Aminotransferase 1 (BCAT1), shown to be relevant in T-cell metabolic reprogramming upon TCR stimulation *via* regulation of cytosolic leucine utilization (Ananieva et al., 2014), and FAS, a member of the TNF-receptor superfamily, with a central role in the physiological regulation of programmed cell death and optimal CD4^+^ T cell expansion (Puliaeva et al., 2008) are all targeted by more than 20 EV-miRNAs (Figure 1D and Supplementary Table 1). Furthermore, the list of miRNAs targeting CCND1, CDK6, VEGFA, IRF4, TFRC, BCAT1, and FAS consistently includes miR-21-5p, miR-16-5p, and miR-155-5p, which are the three molecules with the highest relative expression in Tconv-derived EVs (Supplementary Table 1).

It is also noteworthy that transcripts targeted by a high number of EV-miRNAs, such as CCND1 and VEGFA, were found to be central hubs following a PPI network topological analysis, confirming a potential key role in cellular regulation (Figure 1E and Supplementary Table 2).

### Suppressive Potential of T Conventional Cell-Derived Extracellular Vesicle-MicroRNAs

**FIGURE 2:**
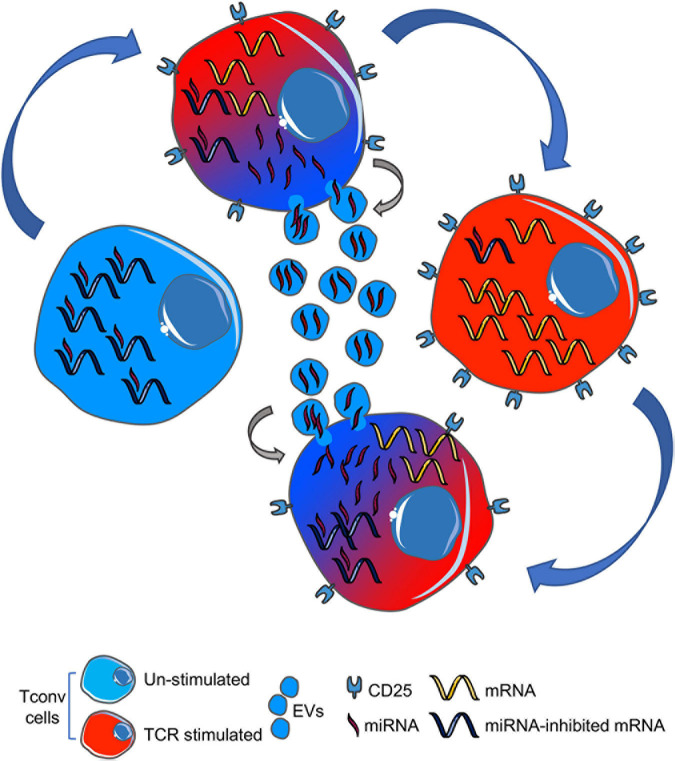
Cartoon summarizing our hypothesis: upon TCR stimulation, a naïve T cell (blue, left) downregulates miRNAs (also) by EV-associated release into the extracellular space (blue to red gradient, upper), thus leading to proper mRNA expression in activated T cell (red, right). EV-miRNAs can be up-taken by an adjacent T cell (red to blue gradient, lower), thus causing mRNA repression and (partial) cell inhibition. TCR, T-cell receptor; EVs, extracellular vesicles.

Based on the observation that miRNAs robustly expressed in Tconv cell-derived EVs show an enriched ability to suppress transcripts upregulated in the same cells upon TCR stimulation, we hypothesize the following mechanism (depicted in Figure 2): in an unstimulated Tconv cell (blue cell, left), most mRNAs linked to cellular activation are also kept repressed through miRNA-dependent translational inhibition. Upon T-cell activation (blue to red cell, up), CD25 is upregulated and miRNAs are lowered also *via* EV-associated release, resulting in specific activation marker upregulation and full initiation of the T effector transcriptional and functional program (red cell, right). If EV-miRNAs are taken up by a surrounding Tconv cell and exert their inhibitory action on transcriptional program therein, then this EV recipient cell will be (partially) restrained in its activation potential (red to blue cell, down).

## Discussion

An RNA-based paracrine signal transmission, dependent on the stability provided by EV protection, is well suited to regulate the space-confined development of an adaptive immune response, such as that occurring in secondary lymphoid organs. Moreover, the peripheral blood up-tick of immune-derived EV-miRNAs during pathological, but also physiological, activation of the immune system may work as an endocrine negative loop aimed at down-modulating the response through the miRNA-dependent inhibition of specific targets in EV recipient cells (de Candia et al., 2013, 2014; Torri et al., 2017). Notably, in both the human and the murine system, EV-miRNAs were shown to directly participate into CD4^+^CD25^+^ T regulatory (Treg) cell-mediated immune suppression, with Treg-derived EV-miR-146a-5p functioning as a cell-to-cell molecular stop signal in Tconv cells (Okoye et al., 2014; Li et al., 2017; Torri et al., 2017). In addition, it has been recognized that a key mechanism underpinning the immunosuppressive potential of mesenchymal stem cells resides in the release of EVs, which have indeed become an attractive therapeutic biological for the treatment of immune-mediated disorders (Gomzikova et al., 2019). Here, we propose that EVs also released by TCR-stimulated Tconv themselves may contain a fingerprint of miRNAs prompted to exert potential suppression activity. Our computational analysis has given support to this hypothesis, showing a significant enrichment of transcripts upregulated upon activation among the targets of Tconv cell-derived EV-miRNAs. Moreover, as EVs carry multiple miRNAs, functionally related transcripts may be suppressed simultaneously, with a resulting enhanced biological effect. To better investigate this effect, the analysis of molecular interactions by means of network models represents a valuable tool for identifying molecules with a key regulatory role, and candidates here selected represent a starting point for future in-depth investigation.

The capability of EV-miRNAs to skew Tconv cell activation makes them new immune-modulatory therapeutic targets in inflammatory and/or autoimmune conditions. On the contrary, although our hypothesis is that Tconv cells release miRNAs to rapidly down-modulate them, we do acknowledge that several EV-associated miRNAs are known to instead be upregulated upon TCR stimulation and to actually activate the Tconv-mediated immune response. A relevant example is that of three members of the mir-17-92 cluster (i.e., miR-19a, 19b, and 20a, among the first 10 most expressed EV-miRNAs, as well as among the miRNAs targeting the highest number of activation-linked transcripts); the cluster is known to sustain lymphocyte proliferation, inhibit cellular death, and push toward a more pronounced pro-inflammatory type-1 phenotype, and hence these miRNAs were proposed as potential targets for the clinical intervention in autoimmunity (Ventura et al., 2008; Xiao et al., 2008; Sasaki et al., 2010; Liu et al., 2014; Wu et al., 2015). Similarly, miR-155, the third most represented miRNA in EVs and the second in terms of transcripts targeted, has also been shown to promote both Th1 and Th17 differentiation and cytokine secretion; an anti-miR-155 treatment has hence also been proposed to reduce Th1/Th17-related inflammation and the autoimmune response (Yao et al., 2012; Yan et al., 2016; Zhang et al., 2017). The design of EV-miRNAs as a novel therapeutic approach should thus contemplate the pleiotropy of their transcriptional regulation and coordinated modulation of tens of transcripts altogether and carefully consider their final biological effect on the “activated T-cell expression network.”

Another important frontier will regard different T-cell subset flexibility in terms of EV-miRNA release. We are now aware of several examples of cytokines that, initially discovered as subset-restricted, are instead produced by functionally distinct T-cell populations (the best example being IL-10, initially discovered as a Th2-type cytokine but actually released also by Th1, Th17, and Treg cells) (O’Shea and Paul, 2010). We already know that different T-cell subsets release specific miRNA fingerprints, yet highly overlap in terms of components, and are not strictly correlated with intracellular expression, indicating regulatory mechanisms behind miRNA disposal *via* EV release (Rossi et al., 2011; Okoye et al., 2014; Torri et al., 2017), but we are still far from fully recognizing the association between EV-miRNA quantitative traits and the actual biological effect. To increase complexity, one miRNA can inhibit different sets of transcripts in different cell contexts; thus, the EV recipient cell may also dictate the EV-miRNA functional output. T-cell-derived EVs were shown to be devoid of coding RNA transcripts, being instead dramatically enriched with small non-coding regulatory RNA molecules (Torri et al., 2017), which suggests that mRNAs may not be a quantitatively relevant part of the EV-associated molecular message. On the other hand, we have taken into consideration exclusively the EV-miRNA cargo, but Tconv-derived EVs do also contain a plethora of other non-coding RNAs, many of which are abundant, evolutionary well conserved, and associated to gene regulatory functions beyond the action of miRNAs (Nolte-’t Hoen et al., 2012). In addition, since Tconv cell-derived EVs have not yet been described to possess proliferative/functional suppressive capacity toward other cells, the “non-miRNA cargo,” including uncharacterized proteins and lipids, may be functionally dominant in natural vesicles, with relevant consequences regarding the design of engineered immune-suppressive EVs. Moreover, another relevant point to be considered is the potential pleiotropy of Tconv-derived EVs in terms of target cell types; notably, the actual passage of miRNAs from T cells to pancreatic β-cells was shown to alter their gene expression asset and cause β-cell death in a murine model of type 1 diabetes (Guay et al., 2019). This study is representative of the general possibility that, *in vivo*, the tissue context can be determinant in dictating not only the function but also the cellular targets of T cell-derived EVs.

## Conclusion

We have here highlighted that miRNAs loaded onto EVs and released by Tconv cells upon TCR stimulation are enriched in molecules suppressing transcripts whose upregulation associates with functional activation; further experiments should thus be designed to test the hypothesis that these EV-miRNAs can indeed produce inhibitory effects in bystander cells. Moreover, in order to use the regulatory function of EV-miRNAs for therapeutic purposes, we will have to pinpoint throughout the relation with other EV components and target transcripts to efficiently curb the function of Tconv cell subsets in pathological conditions such as autoimmunity.

## Materials and Methods

### Human T Conventional Cell Purification and Culture

CD4^+^CD25^–^ T cells were purified from peripheral blood mononuclear cells (PBMCs) from buffy coats of human healthy donors by magnetic cell separation with the Dynabeads Regulatory T Cell Kit (Invitrogen), allowing the separation of a 95–99% pure cell population by FACS analysis. Cells were then stimulated *in vitro* for 72 h in serum-free AIMV Medium AlbuMAX supplement (Gibco) in the presence of anti-CD3- and anti-CD28-coated Dynabeads (0.2 beads per cell) (Invitrogen).

### Extracellular Vesicle Isolation and MicroRNA Profiling

Human Tconv cell-derived EVs were isolated from conditioned media using size exclusion chromatography (Exo-spin^TM^ columns, Cell Guidance) according to the manufacturer’s protocol. The characterization of Tconv cell-derived EVs isolated with such a procedure is reported elsewhere (Torri et al., 2017). Isolated EV eluate was total RNA extracted using miRNeasy serum/plasma advanced kit (Qiagen, United States) and a fixed volume of eluted RNA sample was used as input for reverse transcription reaction by miRCURY-LNA RT Kit according to the manufacturer’s instruction (Qiagen, United States). EV-associated miRNAs (*n* = 752) were profiled by using the complete human miRCURY LNA miRNA panel I + II (V5, Qiagen, United States).

### Microarray Analysis

The list of genes that are either upregulated or downregulated by TCR stimulation in Tconv cells (Supplementary Table 1) was previously obtained by total RNA hybridization onto Agilent Whole Human Genome 4 × 44K, with detectable raw intensities being log-2 transformed, normalized by the quantile method across the arrays and analyzed, as described (Procaccini et al., 2021).

### Bioinformatics Analysis

Experimentally validated miRNA targets were automatically retrieved by miRecords (Xiao et al., 2009), miRTarBase (Hsu et al., 2011), and TarBase (Karagkouni et al., 2018), using an in-house R script based on readxl, xlsx, and multiMiR libraries; score cutoff was set to the default value 20 (search the top 20%). Using these data, a miRNAs–targets network was reconstructed by the Cytoscape platform (Shannon et al., 2003), maintaining exclusively the targets present in the list of transcripts/genes differentially expressed in Tconv cells upon TCR stimulation. The miRNAs–targets reconstructed network was processed at the topological level, by Centiscape Cytoscape’s App (Scardoni et al., 2014), to calculate the node Degree centrality (the number of interactions of each node in the network). Using JMP 15.1 SAS statistical software, the degree distribution of up- and downregulated transcripts was compared by Student’s *t*-test. The list of transcripts differentially expressed following TCR stimulation was also used to reconstruct a PPI network by String Cytoscape’s App (Doncheva et al., 2019); specifically, only Experiments (score >0.0031) and Databases (score >0.36) annotated interaction were considered. The reconstructed PPI network was finally processed at the topological level by Centiscape Cytoscape’s App (Scardoni et al., 2014), to calculate the node Betweenness, Bridging, and Centroid centralities; nodes with centrality values above the average calculated on whole network were considered hubs, as previously reported (Di Silvestre et al., 2021). In addition, statistical significance of topological results was tested by considering randomized network models (Supplementary Figures 1C,D): they were reconstructed and analyzed by an in-house R script based on VertexSort (to build random models), igraph (to compute centralities), and ggplot2 (to plot results) libraries; the results were visualized in the form of violin plots.

## Data Availability Statement

The datasets presented in this study can be found in online repositories. The names of the repository/repositories and accession number(s) can be found below: Gene Expression Omnibus: GSE154401 and GSE183713.

## Ethics Statement

The studies involving human participants were reviewed and approved by Institutional Review Board of the Università degli Studi di Napoli “Federico II.” The patients/participants provided their written informed consent to participate in this study.

## Author Contributions

PdC conceived the original idea and wrote the article. DD developed the investigation workflow, performed statistical analyses, and assembled and critically evaluated the data. GP developed the investigation workflow and performed statistical analyses. SG carried out the microRNA quantification. CP, FP, VD, PM, and GM contributed to the data discussion and draft writing and critically revised the article. All authors approved the final version of the manuscript.

## Conflict of Interest

The authors declare that the research was conducted in the absence of any commercial or financial relationships that could be construed as a potential conflict of interest.

## Publisher’s Note

All claims expressed in this article are solely those of the authors and do not necessarily represent those of their affiliated organizations, or those of the publisher, the editors and the reviewers. Any product that may be evaluated in this article, or claim that may be made by its manufacturer, is not guaranteed or endorsed by the publisher.

## Supplementary Material

Supplementary Table 1 List of transcripts (*n* = 360) found to be either significantly up- (UP) or down- (DOWN) regulated following TCR stimulation. For each transcript, average microarray expression level in stimulated and un-stimulated condition, fold change between the two conditions (logFC), total number and names of miRNAs targeting them are also reported. For miRNAs, the relative quantities of EV-expression level appear in the upper row.

Supplementary Table 2 Hubs from protein-protein interaction (PPI) network reconstructed starting from transcripts either up- or down-regulated following TCR stimulation. Betweenness, Bridging and Centroid centralities were calculated and nodes with centrality values above the average calculated on whole network were considered Hubs. For each transcript, fold change (logFC) and number of targeting miRNAs are also reported.
