# Supplementary material for: CD4+ T-Cell Activation Prompts Suppressive Function by Extracellular Vesicle-Associated MicroRNAs
Source: Front Cell Dev Biol. 2021 Oct 27;9:753884. doi: 10.3389/fcell.2021.753884 (PMC8580371; doi:10.3389/fcell.2021.753884)
Supplement: Supplementary file 3 [file Data_Sheet_1.PDF]

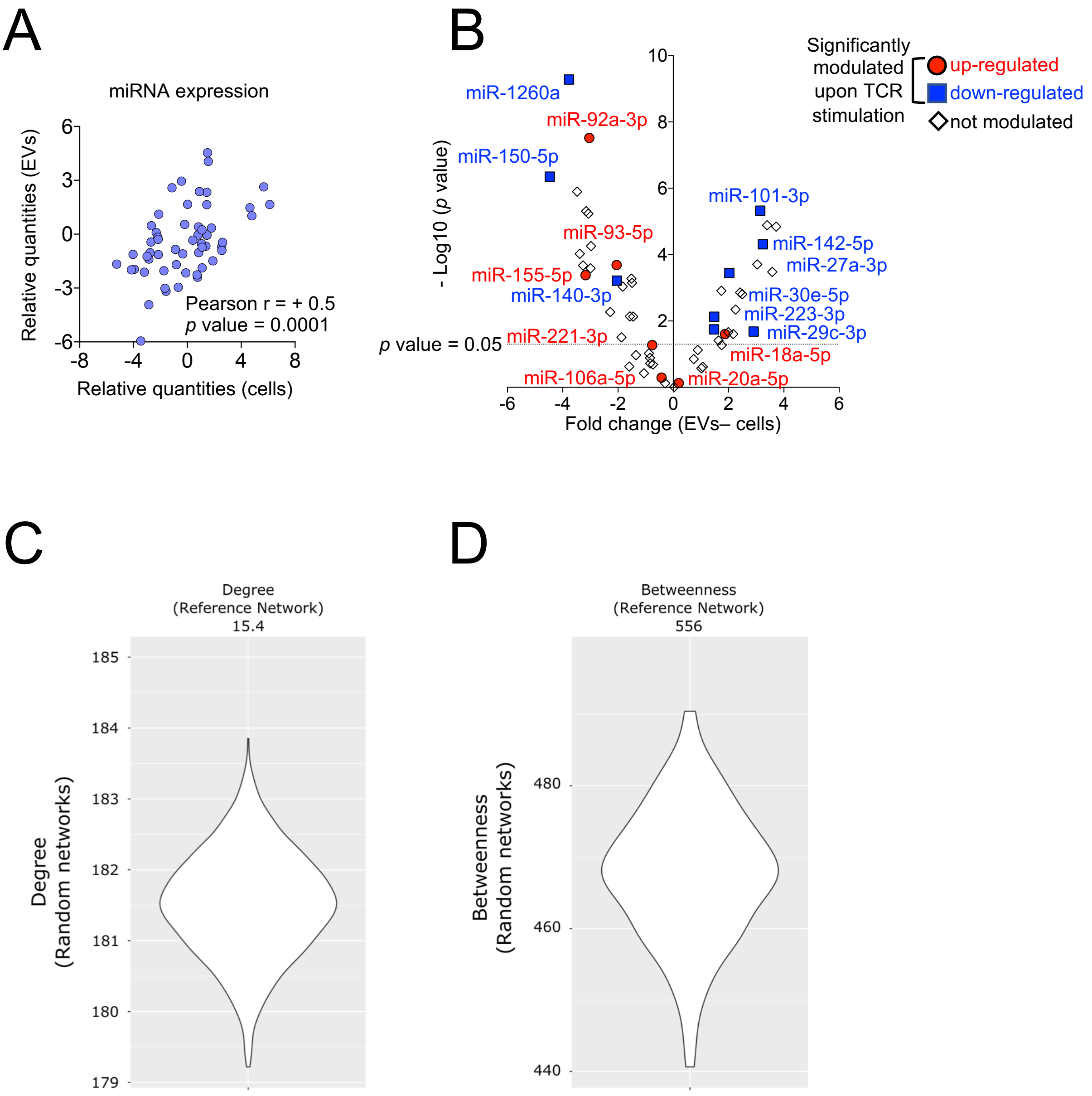

Figure S1. (A) Scatter plot showing the correlation analysis between miRNA relative quantities in EVs and cells. Pearson *r* and relative *p* values are also reported. **(B)** Volcano plot showing miRNA enrichment (right) or depletion (left) in EVs compared to cells with the relative statistical significance. Red and blue colors refer to known up- or down-regulation (respectively) of indicated miRNAs upon Tconv cell activation[15,16]. **(C-D)** Violin plots reporting the average Degree **(C)** and Betweenness (**D**) in miRNA-targets and protein-protein interaction (PPI) random network models, respectively; the average Degree and Betweenness values calculated from reference networks are shown. TCR=T cell receptor; EVs=extracellular vesicles.
